# The Coronavirus Calendar (CoronaCal): a simplified SARS-CoV-2 test system for sampling and retrospective analysis

**DOI:** 10.3389/fepid.2023.1146006

**Published:** 2023-05-23

**Authors:** Manija A. Kazmi, David S. Thaler, Karina C. Åberg, Jordan M. Mattheisen, Thomas Huber, Thomas P. Sakmar

**Affiliations:** ^1^ Laboratory of Chemical Biology and Signal Transduction, The Rockefeller University, New York, NY, United States; ^2^ Biozentrum, University of Basel, Basel, Switzerland; ^3^ Program for the Human Environment, The Rockefeller University, New York, NY, United States; ^4^ Tri-Institutional Program in Chemical Biology, New York, NY United States

**Keywords:** SARS-CoV-2, COVID-19, saliva, virus, epidemiology, public health, RNA, sample preservation

## Abstract

**Objectives:**

To develop a biological diary (CoronaCal) that allows anyone in the community to collect and store serial saliva samples and chart symptoms on ordinary printer paper.

**Methods:**

Diaries were analyzed for the presence of SARS-CoV-2 RNA using established polymerase chain reaction (PCR) procedures. CoronaCal diaries were distributed to volunteer subjects in the community during the peak of the COVID-19 outbreak in New York. Volunteers collected their own daily saliva samples and self-reported symptoms.

**Results:**

SARS-CoV-2 RNA extracted from CoronaCals was measured using qPCR and RNA levels were correlated with reported symptoms. SARS-CoV-2 RNA was detected in CoronaCals from nine of nine people with COVID-19 symptoms or exposure to someone with COVID-19, and not in one asymptomatic person. CoronaCals were stored for up to 70 days at room temperature during collection and then frozen for up to four months before analysis, suggesting that SARS-CoV-2 RNA is stable once dried onto paper.

**Conclusions:**

Sampling saliva on simple paper provides a useful method to study the natural history and epidemiology of COVID-19. The CoronaCal collection and testing method is easy to implement, inexpensive, non-invasive and scalable. The approach can inform the historical and epidemiological understanding of infections in individuals and populations.

## Introduction

Active management and historical analysis of infectious diseases outbreaks depends on preserved biological and bio-cultural samples (1–3). Retrospective analysis of properly documented serial samples can help determine key parameters of pandemic outbreaks. For example, in cases of emerging viral diseases, knowledge about incubation periods, the relationship between transmissibility and symptoms, and variability in the length of time that individuals stay infected could be used to plan and update effective mitigation strategies (4). Rapid real-time testing for SARS-CoV-2 has been prioritized based on urgent and immediate clinical needs (5). However, primary biological samples may be preserved in liquids at room temperature indefinitely, although storage of samples requires significant long-term commitment of resources (6, 7). Isolated nucleic acids are stable, but their purification also requires significant resources. Previous work has shown that nucleic acids are recoverable from biological liquids, including saliva, dried on specialized paper filters and stored at −70°C for up to 14 months and then one week at ambient temperature (8). Saliva is easy to sample and widely used for clinical SARS-CoV-2 real-time quantitative qPCR assays (9–11).

Here, we tested the hypothesis that saliva dried onto ordinary laser printer paper and stored at room temperature or in a household freezer preserves RNA, allowing for later recovery and assay. We designed and implemented a biological diary called CoronaCal to allow for simple daily saliva self-collection in real-life situations in the community. SARS-CoV-2 RNA was transiently detected in volunteers who contemporaneously recorded symptoms over many days, which coincided with virus infection as shown by conventional assays. Human RNase P RNA was used as a positive control and was shown to be stable over several months. The ability to assay SARS-CoV-2 RNA on archive calendars has important potential to enrich epidemiology and thereby contribute to rational implementation of control measures.

## Methods

### Materials and reagents

RNA extraction was carried out using Trizol reagent (Life Technologies, cat#15596026) and the Direct-Zol RNA Microprep kit (Zymo Research, #R2060) using RNase Inhibitor (New England Biolabs, cat#0314S). Additional consumables included AriaMx 96-well plates (VWR Agilent, #401490), adhesive seals (Agilent #401492), optical cover compression pads (Applied Biosystems #4312639) and VacConnectors (Qiagen #19407). RT-qPCR was carried out using the GoTaq Probe 1 Step qPCR system (Promega #A6120) on an Agilent AriaMx qPCR instrument. All primers, probes and control plasmids were from IDT Integrated DNA Technologies. N1 and N2 primer probes were from the 2019 nCoV CDC EUA Kit (IDT #10006606). RNase P Forward primer (IDT #10006827), RNase P Reverse primer (IDT #10006828) and RNase P ATTO 647 probe (IDT #10007061) were used as follows: 20-µl RP forward, 20-µl RP reverse, 10-µl RP probe were mixed with 950-µl Tris-EDTA, pH 8.0 buffer and 2-µl were used per reaction along with 1.5-µl of the N1 and N2 primer probes. Positive control plasmids were IDT 2019 nCoV_N_Positive Control 200,000 copies/ul and IDT 2019 Hs_RPP30_Positive Control 200,000 copies/µl. These were used to make a stock containing 500 copies/µl and 1.5-µl of the stock was used per reaction.

### Sample collection

CoronaCal was designed for use by anonymous, unidentified volunteer participants without counseling. The paper sampling diaries were printed onto Hammermill ForeR Multi-Purpose 20LB white letter-sized printer paper, and the stickers used as symptom log lists were printed on Avery 1″ × 2–5/8″ Rectangle 6460 labels using a Hewlett-Packard Color LaserJet 4700DN printer. CoronaCal templates are available upon request. The materials were packaged along with an instruction sheet into tear-resistant envelopes, which were also used to return the completed CoronaCals. Volunteer participants from the community were asked to prepare a CoronaCal if they had either tested positive on a PCR-COVID-19 test or had been exposed to someone who had recently tested positive. The participants remained anonymous and unidentified, and data obtained from the diaries were not correlated with any information external to what was archived on the CoronaCals themselves. The protocol for the collection of the biological diaries described in this study was reviewed by the Institutional Review Board (IRB) at Rockefeller University and was deemed not to be human-subjects research. CoronaCals were kept at room temperature then returned to the laboratory where they were stored for up to four months at −20°C until assayed.

### Sample processing

CoronaCals were processed as follows: Eight 2-mm-wide × ∼17-mm long paper strips were excised from the oval collection zone for each day on the diary using a concatenated razor blade device, and each strip was transferred into RNase/DNase-free 1.5-ml tubes. We estimate that the equivalent of about 3–5 µl of saliva was present on each strip of paper cut from the sample application oval. Each sample was incubated in 300 µl of Trizol for 15 min at room temperature while shaking at 500 rpm. Next, RNA from the sample was purified using the Zymo Direct-zol RNA microprep kit following manufacturer's protocol and eluted with 15 µl of DNase/RNase-free water containing RNase inhibitor (17 units).

### RT-qPCR reactions

Promega's GoTaq Probe 1 Step qPCR reactions were set up in duplicate in 96-well plates using 3-µl of template with either N1 or N2 primer probes from IDT 2019 nCoV CDC EUA Kit along with the RP primer set and ATTO probe using the following cycling conditions: Reverse transcription (one cycle of 15 min at 45°C), hot start (one cycle of 2 min at 95°C), amplification (45 cycles of 3 s at 95°C, 30 s at 55°C). Each plate had a multiplex control reaction containing 2,000 copies each of IDT 2019 nCoV_N_Positive Control and IDT 2019 Hs_RPP30_Positive Control. Data were recorded for up to 45 PCR cycles to give a maximum cycle threshold (Ct) of 45 on data plots. The limit of detection and linearity of the SARS-CoV-2 virus RNA assay were tested using the control plasmids IDT 2019 nCoV_N_Positive Control and IDT 2019 Hs_RPP30_Positive Control. Plasmids were serially diluted from 100,000 copies per reaction down to a nominal value of 0.1 copies per reaction in water and qPCR reactions were set up in triplicate and performed as described above to determine the Ct values for each dilution.

## Results

**Figure 1:**
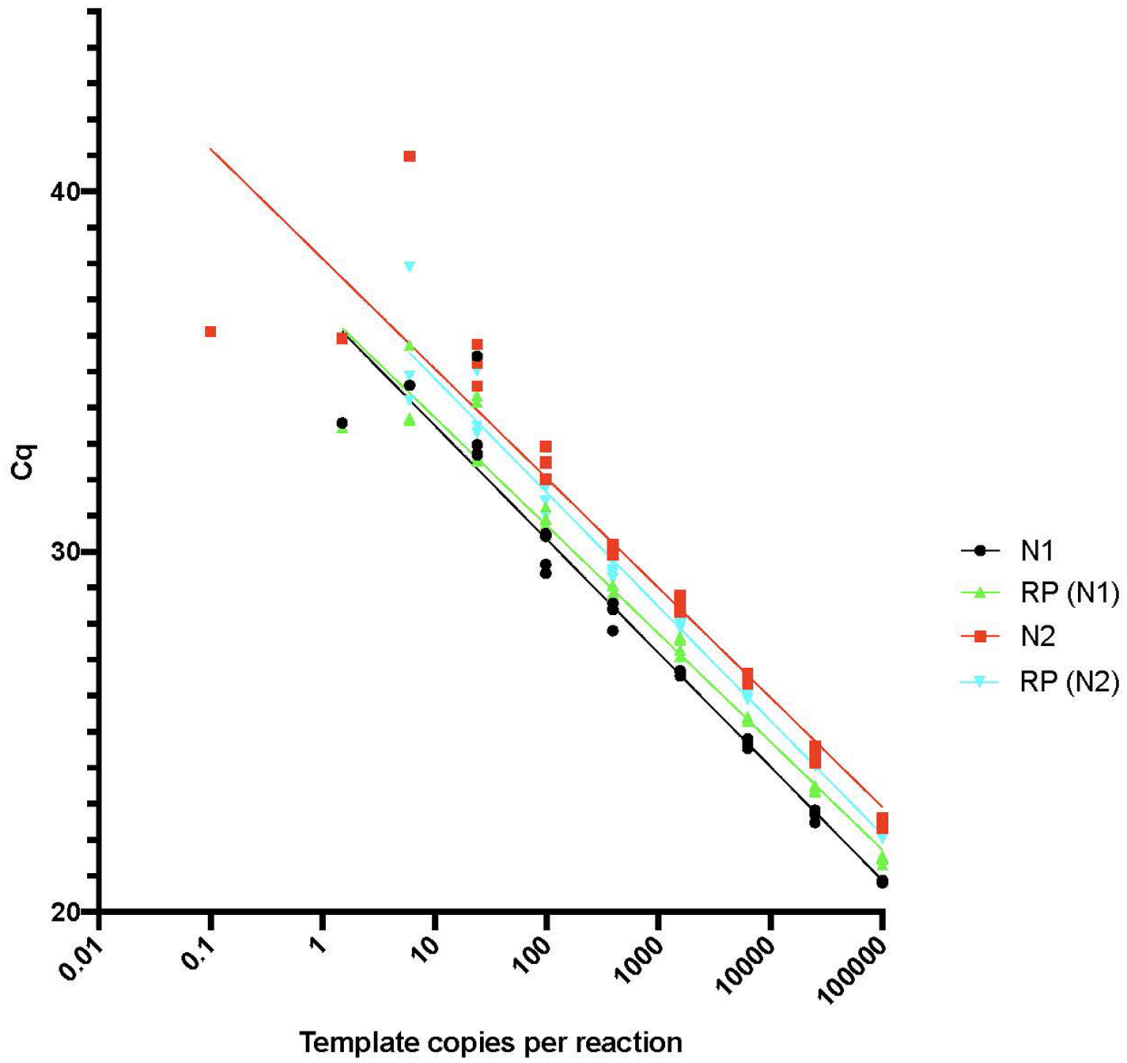
RT-qPCR sensitivity plot. The limit of detection and linearity of the SARS-CoV-2 virus assay was tested using the control plasmids IDT 2019 nCoV_N_Positive Control and IDT 2019 Hs_RPP30_Positive Control as described in Methods. Plasmids were serially diluted from 100,000 copies/reaction down to a nominal values of 0.1 copies/reaction in water and RT-qPCR reactions were set up in triplicate and performed as described to determine the Cq values for each dilution.

The sensitivity and linearity of the RT-qPCR-based detection of the RNaseP control and SARS-CoV2 RNA were measured (Figure 1). RNase P and SARS-CoV-2 RNA signals were reproducibly detected from multiple samplings after different durations of collection with storage at room temperature for up to 44 days. No signal diminution was seen when testing samples stored for different durations after collection at −20°C or after multiple freeze-thaw cycles. These results suggest that the RNA in the dried saliva samples was stable for weeks at room temperature and indefinitely at −20°C. Nucleic acids were extracted from CoronaCals and assayed using qPCR. Duplicates were in close concordance, and in the few cases where only one of two of the replicates gave a positive signal, it was still recorded and included in the data plots.

**Figure 2:**
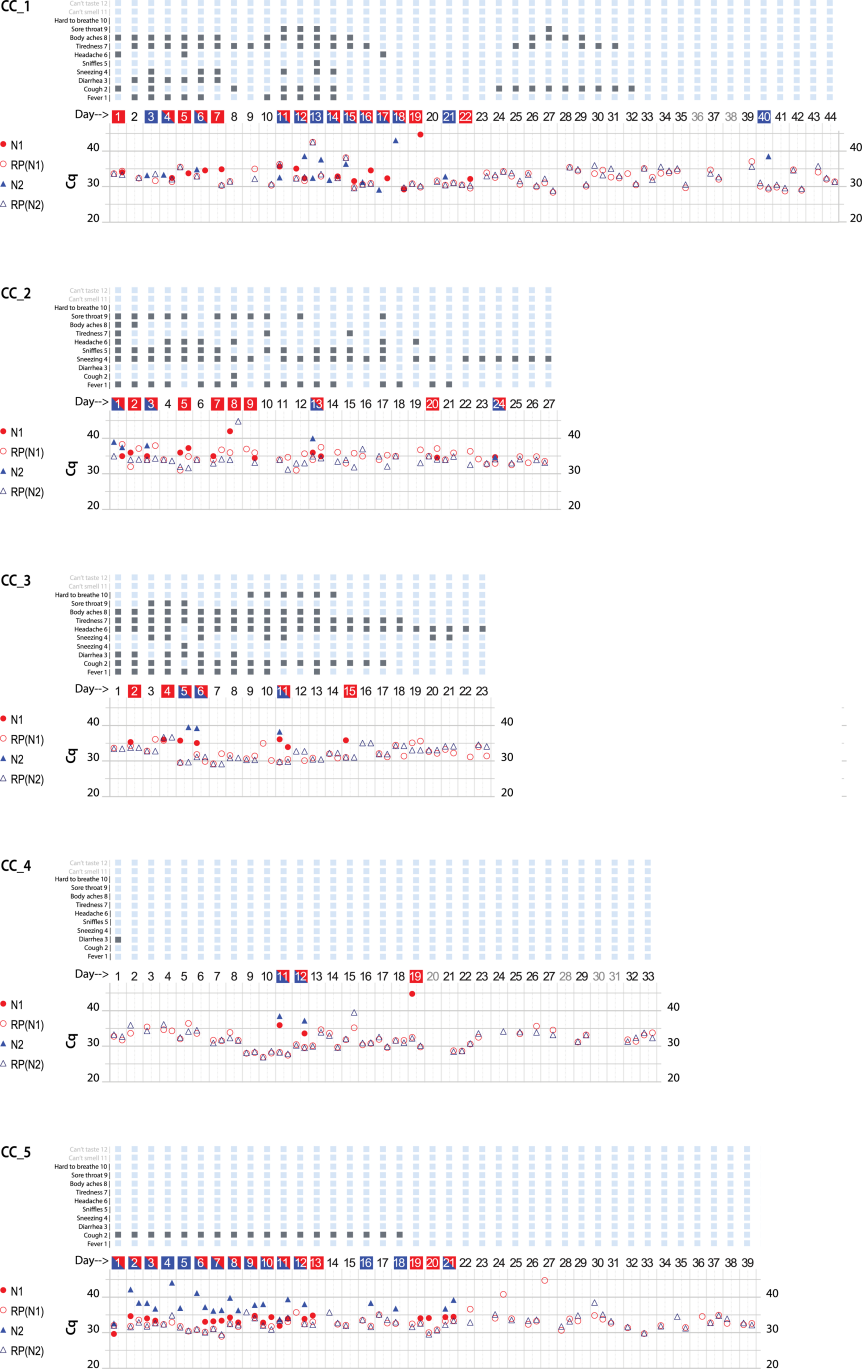
Graphical presentation of data obtained from ten CoronaCal diaries. The ten CoronaCals presented are labeled CC_1 through CC_10. For example, CC_1 shows data from a CoronaCal diary in which one individual collected saliva samples and self-recorded symptoms for 44 consecutive days during the course of a COVID-19 illness. Each chart is essentially a timeline that can be read from left to right. Each column demarked by vertical grey lines represents one day. At the top of the figure, filled boxes represent self-reported symptoms: (1) fever; (2) cough; (3) diarrhea; (4) sneezing; (5) sniffles; (6) headaches; (7) tiredness; (8) body aches; (9) sore throat; (10) hard to breath; (11) can’t smell; (12) can’t taste. The bottom section of the chart shows the results of qPCR analysis of RNA samples extracted from the paper CoronaCal. Duplicate samples were analyzed for each day using PCR primer/probe combinations designed to detect SARS-CoV-2 N1 (red solid circles) and N2 (blue solid triangles). For each sample, and for each N1/N2 probe, saliva RNAase P RNA was measured as a control (open circles and triangles). The day numbers are boxed in red when the PCR threshold for N1 positive was reached, blue if the Cq threshold for N2 positive was reached, or both red and blue when both N1 and N2 were deemed to be positive. An absence of symbols indicates that no amplicon was detected after 45 PCR cycles, and days in light grey indicate days where no sample was collected.

CoronaCals were analyzed from ten different individual anonymous volunteers (Figure 2). CoronaCals 1 (CC_1) and 7 (CC_7) included samples from 44 consecutive days (with the exceptions of a few blank days on CC_1). The control signal of human RNase P was consistent across the entire time series in these samples, serving as a check for the assay procedure, including the extraction and qPCR steps. Importantly, these results show the RNA moiety of human RNase P was stable under these conditions because the quantification PCR cycle threshold (Cq) does not increase across the sample. If RNA target degradation were occurring on the stored CoronaCal, one might expect that samples earlier in the series would have less signal than those later in the series. Samples collected on day one were stable *in situ* and at room temperature on the CoronaCal for between 5 and 6 weeks. Detection of RNase P was reliable and continuous over the entire period showing the stability at room temperature of the RNA signal under these conditions.

**Figure:**
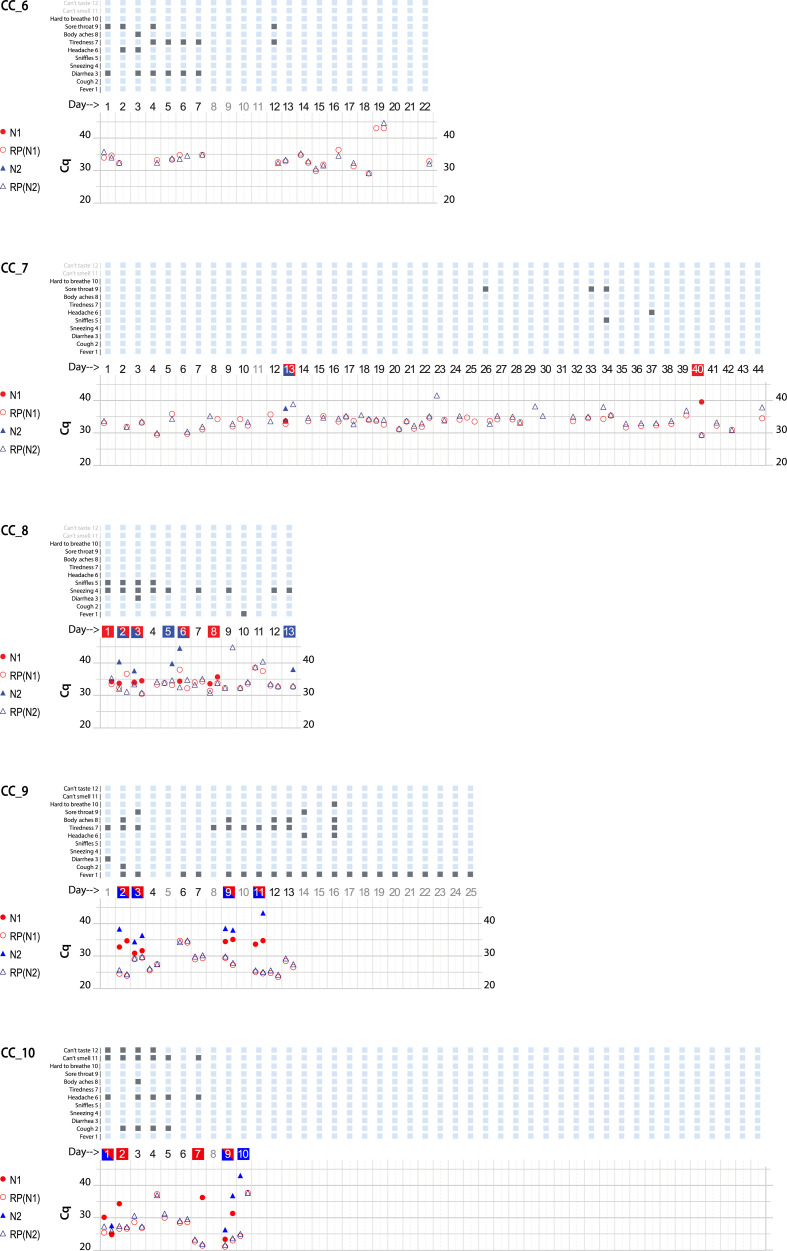


CoronaCals CC_1 through CC_8 were collected from participants in New York City during March through June 2020 when the original Wuhan SARS-CoV-2 was prevalent. CC_9 and CC_10 were collected later when the so-called Alpha variant was dominant. All ten CoronaCals were collected before COVID-19 vaccines were available and none of the participants were vaccinated. The underlying health status or COVID-19 risk factors are not known for any of the anonymous and unidentified participants.

Each CoronaCal reports the course of one person's COVID-19 illness. For example, on Day 1 of CC_1, the subject reported cough, headache and body aches and was also positive for SARS-CoV-2 RNA (N1 only). Fever appeared on Day 2 along with other symptoms. On Day 3 through Day 7, the positive saliva RNA persisted and symptoms evolved. By Day 8 of the illness, some symptoms had subsided and there was no longer SARS-CoV-2 RNA present in the saliva. However, after three consecutive days with fewer symptoms and no detectable SARS-CoV-2 RNA, fever recurred on Day 11, as did the appearance of SARS-CoV-2 RNA. On Day 18 of the illness, the subject was asymptomatic, but continued to be positive for saliva RNA for four out of the next five days.

SARS-CoV-2 positive qPCR signals, were transient and discontinuous, consistent with the natural course of infection as seen in studies that conducted standard qPCR assays each day (12). None of the nine CoronaCals with positive results for SARS-CoV-2 RNA detection had continuous daily positive tests, became negative and then remained negative. All the positives were discontinuously positive. The participant who was positive for SARS-CoV-2 RNA for the longest duration was CC_5, who was positive for 13 consecutive days. CC_5 was also positive for 18 of the first 21 days of illness. CC_5 reported only cough, but for 18 consecutive days, and also remained positive for SARS-CoV-2 RNA even after becoming asymptomatic. The duration of positive tests was striking in CC_2, which showed positive tests for ten days over a span of 24 days (see also CC_1 and CC_5). One caveat is that not all testing detected both N1 and N2.

The stability of RNA signal is evident in the consistency of RNase P control detection. For example, from CC_1 the control RNA Cq values are not significantly different on Day 1 than on Day 44 (the average Cq = 32.7 ± 2.4 for the RNase P control signal). Even though Cq values were consistent for a given CoronaCal, they did seem to vary when averages were compared among the ten CoronaCals tested, suggesting that individuals dabbed different amounts of saliva, but each individual was consistent in how much they dabbed from one day to the next. The Day 1 sample for CC_1 was kept at room temperature for at least 44 days before it was placed at −20°C for long-term storage. Day-to-day Cq controls are affected by the amount of saliva applied, which is not measured or so controlled, and is therefore expected to vary.

## Discussion

CoronaCals were made from ordinary printer paper with designated spaces to place a fingertip's worth of saliva (∼30–50 µl) each day. The saliva spot was allowed to air dry, then covered with an adhesive label. The covering label serves two functions: (1) it isolates samples preventing cross contamination, (2) it decreases the already very small chance of fomite transmission (13) or aerosolization from dried saliva. The covering sticker also allows space for recording symptoms and could conceivably be used for a barcode that could be coordinated with cellphone apps for location, proximity to others, and analysis pipelines. The paper preservation format could be implemented by individuals or employed in a coordinated way for groups traveling, working, or living together.

The relationship between self-reported symptoms and SARS-CoV-2 RNA signal is of interest. Large studies have shown that positive PCR tests are not always associated with symptoms and conversely, that symptoms can occur during the absence of a PCR signal (12). The CoronaCal data demonstrate directly this phenomenon during the time courses of infections in individuals. The CoronaCal method relies totally on self-reporting and self-collection. The study team had no direct contact with the volunteer participants to instruct or influence their use of the diaries or to attempt to increase compliance. Although participants were provided with enough materials to collect up to 84 days of samples, the longest diaries collected were for 44 days. The shortest diary collected was for 10 days. Compliance is an issue for any self-collection and reporting system. Although compliance varied, a tremendous amount of information could be obtained if the strategy was scaled-up to larger numbers of participants. In addition, more information could be obtained if participants could be interviewed, or if everyone in a particular household, for example, completed a CoronaCal diary contemporaneously. Such a study would require informed consent, however, and was not our aim for a proof-of-concept pilot study to develop and validate the methodology.

A key finding of this work is that signals for SARS-CoV-2 as well as human RNase P were stable for extended periods in small samples of saliva dried onto ordinary printer paper. The stability of RNA signals found in this study is conceivably due to special features of the RNAs involved. SARS-CoV-2 RNA packaged inside virions may be protected from nucleases and/or otherwise stabilized (14). It is not known from this work, how much of the SARS-CoV-2 signal detected in saliva is present in packaged virions, and how much is inside epithelial cells or cellular debris that have been shed into saliva. RNase P RNA is normally part of a large multi-subunit complex, and this complex might shield the RNA signal (15, 16). At this time, the stability of other RNA moieties in this format, such as mRNA, is unknown. Our hypothesis is that rapid drying saliva samples onto a porous paper surface is the key factor responsible for signal stability. Previous studies have shown that SARS-CoV-2 RNA is detectable for extended periods on surfaces (17). The present work exploited this observation to develop a format for non-invasive, resource-sparing sample archiving, extends the sampling period, and uses ordinary printer paper. In addition, the distinction between detectable RNA and infective virus is important to keep in mind, since virions can be rendered non-infectious before RNA becomes non-detectable (18).

One goal of this study was to find the right balance between reproducibility and ease-of-use to design a convenient user-friendly saliva collection that could be used to preserve personal daily saliva samples along with self-reported symptoms. Saliva samples, dried down onto a calendar printed on ordinary printer paper becomes a “biological diary” that can support molecular analysis weeks or months after the samples have been deposited. The system is inexpensive and scalable, and in principle can be deployed without the need for cumbersome infrastructure. Although a detailed cost-benefit analysis is beyond the scope of the present pilot study, we believe that the CoronaCal platform could be used economically in a variety of scenarios and contexts to provided added value in the surveillance and response to viral outbreaks.

Most nucleic acid recovery from non-optimal and ancient materials has been DNA (19). However, the recovery of RNA sequences from the 1918–1919 influenza via paraffin sections and victims exhumed from permafrost graves (20) and HIV from lymph node embeds from 1960s (21) shows that RNA under at least some circumstances is more stable than often assumed. Archived samples have previously proven important in tracing the origin and spread of infectious diseases. However, in the cases of which we are aware, materials were originally collected for other purposes and never systematic time series. Molecular analysis used to study the origin of HIV did not exist at the time samples were taken (21).

Discovery approaches such as untargeted sequencing and other “omics” have the potential to identify novel infectious agents and biomarkers beyond those that have been characterized at the time of sample collection (22). Future analytical methods developments should increase the value of archived biological samples, which likely contain more potential information than can currently be interpreted (23). In addition to records of individuals, related approaches would be valuable if applied to wastewater and eco-environmental contexts (24, 25). Archived biological diaries from multiple levels would add rich information to retrospective reviews aimed at improving pandemic response (26), public health more generally (27) and enhancing understanding of our evolving environment (19, 28, 29).

## Data Availability

Digital templates of the CoronaCal diary designs in various formats are available upon request.
